# The considerations, experiences and support needs of family members making treatment decisions for patients admitted with major stroke: a qualitative study

**DOI:** 10.1186/s12911-020-01137-7

**Published:** 2020-06-01

**Authors:** A. Visvanathan, G. E. Mead, M. Dennis, W. N. Whiteley, F. N. Doubal, J. Lawton

**Affiliations:** 1 Clinical Academic Fellow (Chief Scientist Office), Centre for Clinical Brain Sciences, The University of Edinburgh, 49 Chancellor’s Building, Edinburgh, EH16 4SB UK; 2 Usher Institute, The University of Edinburgh, Teviot Place, Edinburgh, EH8 9AG UK

**Keywords:** Major stroke, Decision-making, Family members, Experiences, Needs

## Abstract

**Background:**

Treatment decision-making by family members on behalf of patients with major stroke can be challenging because of the shock of the diagnosis and lack of knowledge of the patient’s treatment preferences. We aimed to understand how, and why, family members made certain treatment decisions, and explored their information and support needs.

**Method:**

Semi-structured interviews with family members (*n* = 24) of patients with major stroke, within 2 weeks of hospital admission. Data were analysed thematically.

**Results:**

Families’ approach to treatment decision-making lay on a spectrum according to the patient’s state of health pre-stroke (i.e. patient’s prior experience of illness and functional status) and any views expressed about treatment preferences in the event of life-threatening illness. Support and information needs varied according to where they were on this spectrum. At one extreme, family members described deciding not to initiate life-extending treatments from the outset because of the patients’ deteriorating health and preferences expressed pre-stroke. Information from doctors about poor prognosis was merely used to confirm this decision. In the middle of the spectrum were family members of patients who had been moderately independent pre-stroke. They described the initial shock of the diagnosis and how they had initially wanted all treatments to continue. However, once they overcame their shock, and had gathered relevant information, including information about poor prognosis from doctors, they decided that life-extending treatments were no longer appropriate. Many reported this process to be upsetting and expressed a need for psychological support. At the other end of the spectrum were family members of previously independent patients whose preferences pre-stroke had not been known. Family members described feeling extremely distressed at such an unexpected situation and wanting all treatments to continue. They described needing psychological support and hope that the patient would survive.

**Conclusion:**

The knowledge that family members’ treatment decision-making approaches lay on a spectrum depending on the patient’s state of health and stated preferences pre-stroke may allow doctors to better prepare for discussions regarding the patient’s prognosis. This may enable doctors to provide information and support that is tailored towards family members’ needs.

## Background

Some treatment decisions need to be made early after a major stroke. Treatments such as hemicraniectomy [1] enteral tube feeding [2] and intermittent pneumatic compression [3] increase the likelihood the patient will survive but with significant disability. However, declining these treatments may increase the likelihood of death. Treatment decision-making following major stroke is particularly challenging because most patients do not have the mental capacity and/or are too medically unwell to understand the consequences of treatments. To ascertain patient preferences in these situations, professional organisations such as the General Medical Council and The American College of Critical Care Medicine encourage doctors to involve proxies (often family members) in decision-making [4, 5]. This recommendation is supported by literature which suggests that families know patients’ preferences the best, [6–10] and seeking patients’ preferences from others is a way of extending patients’ autonomy [11, 12]. Furthermore, patients generally want their family members to be involved in decision-making [13] and most families want to be involved [14–16]..

However, making decisions concerning life and death is not easy for families for several reasons.

Firstly, family members may not know the patient’s preferences and hence they may make decisions based on their own values rather than those of the patient [17, 18]. They may also find it difficult to make decisions that are potentially not life-extending, even if these are consistent with what patients may have previously expressed [19]. Patients may also change their views regarding the acceptability of treatments and potential outcomes once they are faced with a situation of critical illness or significant disability [20–22]. Therefore, family members are faced with two challenges; first, to make decisions based on the current situation; and, second, to predict how the patient may react if left significantly disabled.

Secondly, families may be in shock and making treatment decisions under these circumstances may be overwhelming. This challenge has been reported in various contexts, including in intensive care and severe stroke settings [5, 23, 24].

Thirdly, an important step in facilitating decision-making is for doctors to provide necessary information to families [4, 25, 26]. In the early period after a major stroke, families may be distressed. Hence, too much information may be overwhelming [27] and, families may want information that is specific to their situation along with support from doctors to make treatment decisions [28–30]. Recognising the information and support needs of families in the context of a major stroke is crucial to doctors who may need to tailor their communication to help families make decisions [31–34]..

A mixed methods study has acknowledged the need for effective communication of prognosis and psychological support for family members in the context of dealing with consequences of severe stroke [35]. Literature in stroke have also indicated that family members who make decisions on behalf of the patient wish for information on prognosis [36, 37]. However, to our knowledge, there is a lack of research exploring how and why family members make certain treatment decisions in the early period after a major stroke.

Thus, in this qualitative study, we aimed to address gaps in research on treatment decision-making by family members of patients admitted with major strokes. Specifically, we explored how family members made decisions regarding treatments given in the early period after a stroke that may increase the likelihood of the patient surviving longer, but with significant disability. We explored the factors considered by family members when deliberating about treatments, their early experiences in hospital when these decisions were made and their information and support needs. Based on our results, we provide recommendations for doctors communicating with family members of patients with major stroke in hospital.

## Methods

### Study design

We used semi-structured interviews informed by a topic guide to allow flexibility for participants to discuss issues and experiences which were important to them, including those unforeseen at the study outset and ensure the discussion remained relevant to addressing the study aims [38]. Based on reviews of the literature and discussion with clinical colleagues, we developed a topic guide (Table 1) which allowed us to explore, with families, what the patients’ lives were like before the stroke and how this may have influenced their views and approaches. We also explored their experiences soon after the patient’s admission to hospital in order to set the context for understanding the treatment decisions which were made. Data collection and analysis took place concurrently, enabling issues identified in early interviews to inform areas explored in later ones [39]..

**Table 1:** Topic guide

Interview time	Topics explored
Initial	• How family members saw the patients’ life before the stroke; how patients were coping and if they required any help for their day to day activities. The patients’ previous medical illnesses and experience with health care. • Whether patients had made any pre-stated wishes about treatments in the event of a critical illness and if so, the context in which these wishes were stated. • The emotional reactions of family members to stroke diagnosis and their initial experiences in hospital; if and how they reacted to, and came to terms with, the diagnosis and potential poor prognosis • The factors considered by family members when decisions needed to be made on treatments that were life-extending, but may leave the patient with potentially significant disability; how they arrived at a decision, and why • Based on family members’ experiences in hospital, their early needs; how and why information or support may be useful to them, whether these changed over the first 2 weeks in hospital and if so, how and why.

Interviews took place within the first 2 weeks of the patient’s admission to hospital with a major stroke. This time point was chosen as we wished to capture the early experiences of family members when making treatment decisions. Furthermore, we recognised that most treatments we were interested in exploring (i.e. hemicraniectomy, enteral tube feeding, intermittent pneumatic compression, antibiotics and parenteral fluids) should have been discussed, and decisions made, during this time.

### Recruitment and sampling

We recruited adult family members of patients admitted with major stroke to a large teaching hospital in the United Kingdom. This hospital recruits an average of 500 people a year who are physically disabled as a result of stroke. Around 200 of these patients would be significantly disabled and over 80% of these patients would not have mental capacity. Over 95% of patients are ethnically white, and over a third above the age of 75. To be eligible for our study, the patient needed to be significantly disabled as a result of the stroke and not have mental capacity to participate in decision-making. We defined these patients as having had a major stroke. Treatments (such as hemicraniectomy, enteral tube feeding, parenteral fluids, antibiotics or intermittent pneumatic compression) also needed to have been discussed and decisions made between the doctor and family member.

The medical team identified eligible patients. They considered whether the family member would be appropriate to approach regarding the study (i.e. participation would not be too distressing for them) and, if considered suitable, they asked them if they would be interested in taking part. Where family members were agreeable to being approached, the researcher (AV) then provided them with further information and if family members agreed to take part, AV then obtained informed written consent. AV is a clinical doctor with a Bachelor of Medicine degree (MbChB) specialising in geriatric medicine. Based on her clinical background, she has an interest in improving the involvement of family members in decision-making. She has previously worked in the stroke unit but not during the study duration or the year preceding commencement of this study. Participants were not informed about AV’s clinical background or personal goals.

Recruitment continued till data saturation was achieved; that is when no new findings were identified in new data collected. Interviews were conducted in a private room in the ward where the patient was admitted at a time convenient to the family member. These interviews took place between May 2017 and November 2017 and lasted 20 to 55 min. All interviews were digitally audio recorded and transcribed in full. Table 1 summarizes the main areas explored in these interviews.

### Data analysis

AV (who received formal training in qualitative methods including analysis) and JL (a very experienced non clinical qualitative researcher) analysed the interviews thematically using the method of constant comparison [39]. Both inductive and deductive approaches were used, which allowed unanticipated themes to emerge from data as well as identification of material needed to address the study aims. JL and AV read the interviews repeatedly and cross compared them to identify issues and themes that cut across different individuals’ accounts. Upon discussion and agreement, a coding frame was developed that captured key themes.

We further analysed coded datasets to develop more nuanced interpretations of the data and identify illustrative quotations. We used a qualitative analysis software package (Nvivo version 11, QSR International Pty Ltd.) to facilitate data coding and retrieval.

### Ethics approval

The study was approved by Scotland A Research Ethics Committee (Ref: 17/SS/0029).

To maintain anonymity, participant numbers are used below. For example, FM01 is used to indicate family member 1 and P01 is used to indicate patient 1.

## Results

We interviewed 24 family members. Demographic information and relevant patient data is presented in Table 2.

**Table 2:** characteristics of family members (participants) and the patients

Characteristics of family members (participants) ***n*** = 24	
Mean age in years (range)	62 (32–75)
Gender	8 male, 16 female
Relationship to the patient	3 partners, 19 children, 2 others (cousin, sister)
Ethnicity	All British white
Occupation	13 retired from work, 11 still working
**Characteristics of patients (*n*** **= 24)**	
Mean age in years (range)	85 (55–101)
Gender	7 male, 17 female
Occupation	22 retired, 2 working
Functional status prior to the stroke	11 independent, 13 required care (either a package of care at home or in a care home)
First stroke	23
Had community do not resuscitate order (DNAR)	7
Had pre-existing major comorbidities including dementia, heart failure and renal failure	11

Family members’ decision-making regarding treatments on behalf of the patient admitted with major stroke lay on a spectrum. At one extreme, family members (the majority) described deciding not to initiate treatments from the outset. In the middle of the treatment decision-making spectrum, were family members who initially asked for all treatments to continue but later decided that life-extending treatments were no longer appropriate. At the other end of the spectrum were family members who wanted all treatments to continue at all costs.

Below, we will consider the factors determining these different decision-making approaches. We will then explore how the different approaches adopted by family members seemed to influence their early experiences in hospital, and their accompanying information and support needs. Where possible, we will report our findings based on where family members were on the treatment decision-making spectrum we have identified.

### Reflecting on patients’ health pre-stroke, and preferences for life-extending treatments

#### Family members who had decided not to initiate life-extending treatments

Family members at one end of the treatment decision-making spectrum described how the patient who had been admitted to hospital, many of whom in their 80s’ or 90s’, already had chronic progressive conditions (e.g., dementia and arthritis) prior to their stroke. They described how, over the years, these conditions had resulted in gradual decline in their health and quality of life. Hence, family members noted how these patients had not been fully independent prior to stroke and how some had either lived in a care home or had been reliant on others for aspects of their care, such as washing and dressing. Family members further noted how this dependence on others had been a source of frustration and distress to the patient.

For example, FM01, the family member of P01 noted how P01 had various chronic medical conditions including arthritis and heart disease, and although had lived at home, had needed carers to come in four times a day. FM01 also described how P01’s dependency on others had led to P01 being unhappy with life and extremely low in mood:

‘*P01’s depressed … every time I go up P01’ll say to me I don’t want to be here, [name removed]. I seem to get it every week In fact...... P01 had said to me I love you but I want you to put the pillow over my head…’ (FM01).*

According to these family members, which included FM01 and FM02 (who is quoted below), patients’ increasing frailty and dependence on others had meant that, in many cases, they had indicated their preference, either to their family or their doctor, for not wanting their already poor quality of life to be extended:

*‘Well, P02 has been very unwell for the last nine months now. P02 had caecal carcinoma, so we have been involved with the hospital for a long time. So, we have had all the discussion about, what interventions P02 would want, so...I was in no doubt about what P02 wanted, which is not much.’ (FM02).*

Many of these family members also reported how the patient had thought ahead to a circumstance where a decision might need to be taken regarding resuscitation:

*‘P03 already has a DNR in place. PO3’s a very strong [gender removed].. P03 knew...well told us this is what...if it comes to a point where all the numbers stack up against P03, and finds requiring a DNR, which P03 wants, that would be the line to take. Do not resuscitate’. (FM03).*

#### Family members who had decided to withdraw life-extending treatments over time

Family members who were in the middle of the treatment decision-making spectrum described how, although the patient had generally been quite old (late 70s’ or early 80s’), they had been determined and able to maintain moderately independent lives. This included FM04 who described how P04, in [gender removed] 80s’, had continued to lead a busy and active life right up to P04’s stroke:

‘*When we got to 80, and [name removed] retired; well, P04 continued to work whenever P04 got the chance – P04 couldn’t retire – and what P04’s done since P04 was 80 is chopped wood and split logs … and even on Sunday, the day before this, P04 was working splitting logs. So P04 was very, very active and very strong’. (FM04).*

FM05, likewise, described how P05 had been very determined and, despite having had multiple health problems and hospital admissions, had only needed minimal help to live independently:

*‘Well, P05’s physically very strong, mentally very strong and P05’s had things before which P05’s come back from, in the hospital, heart attacks and quadruple bypass surgery and so on and P05’s quite tenacious about life in general. We just do some shopping and cleaning for P05.’ (FM05).*

In keeping with their relative independence, family members noted how they felt that the patient had not generally thought about a circumstance where they may be left significantly disabled in any meaningful way. Hence, as FM06, the family member of P06 in [gender removed] 80s’ noted, any comments the patient had previously made which had alluded to treatment preferences could not necessarily be interpreted as their true preferences, because they felt that these individuals had not properly considered a future situation of critical illness and/or significant disability:

*‘P06’s friend had a stroke and went into a home … and that allowed me to introduce the subject of what would you like to do in the long term if you weren’t able to live in your own home? And P06’s response was, oh, I’ve never really thought about it. But well, if I couldn’t stay in my own home I’d probably want to come and live with you. But I said that won’t be possible as I work full time, and P06 says ‘oh well, I’d go to a home then.’(FM06).*

#### Family members who had asked for all treatments to continue at all costs

The minority of family members at the other end of the treatment decision-making spectrum reported how the patient had been relatively young and independent prior to their admission. Hence, family members reported that they did not feel that these individuals had considered a situation of critical illness and, therefore, they were not aware of them having articulated their own wishes for treatments in a situation where they might be left significantly disabled. This included FM07 the family member of P07 in [gender removed] 50s:

‘*Not really something that P07 would speak about; like, we like to get away every now and again, sort of, just we go camping and stuff like this; we’ll walk at weekends. It’s not really something that … I don’t think P07’s thought about the, sort of, long term’. (FM07).*

### Early hospital experiences and accompanying needs

#### Family members who had decided not to initiate life-extending treatments

Family members of patients who had already been physically dependent before the stroke, described how these patients had had multiple previous hospital admissions and therefore, how these previous experiences had made it easier for them to understand and accept that the patients’ prognosis might be very poor. For instance, FM08, the family member of P08 in [gender removed] 90s’ who had had a previous stroke, described how FM08 was familiar with being in hospital and was accepting of the fact that P08 was very unwell and might not survive:

‘*I mean, we kind of predicted that this was maybe the way it was going to go with this second stroke P08’s had, the second time P08’s been here; so there’s a bit of history, so it’s easier for all of us to understand the predicament we’re in’. (FM08).*

Given these experiences, and their confidence in knowing what the patient would have wanted with respect to life-extending treatments, these family members reported how they had determined that initiating such treatments would not be in the patient’s best interests. This included FM09 who described how he had considered P09’s preferences and had concluded that the situation P09 was now in (significantly disabled and requiring 24 h care) would not be the kind of life P09 would want to endure:

‘*P09 said P09 did not want people looking after [gender removed] and I think the point with P09’s situation is that massive stroke – it’s unlikely P09 will recover from it. If P09 does recover … P09’s going to need full-time care, so that’s … for P09 that’s not an option; P09 wouldn’t want that.’ (FM09).*

These family members thus described how they had already decided not to initiate life-extending treatments even before the doctor had provided their opinion on the patient’s prognosis. Hence, as FM10, the family member of P10 in [gender removed] 90s’, described, a discussion with the doctor was often used to justify a decision that had already been made, rather than to arrive at a decision:

‘*So, we’ve (referring to FM10, P10 and family) been very open about it and feel very strongly that no prolonging of life, given the quality of life that P10 has. So, that was the conversation I had with the consultant and it was rather nice and refreshing that the consultant was very open to listening and in total agreement with that, and also being quite honest as to the implications of the stroke, in terms of swallowing and the options, and things like that’. (FM10).*

#### Family members who had decided to withdraw life-extending treatments over time

Family members of patients who had been moderately independent and had not formally expressed their preferences for life-extending treatments, described having been shocked and distressed by the diagnosis of a major stroke with poor prognosis. This included FM11 who shared [gender removed] astonishment at how, on the same day as the stroke, P11 had been leading a group tour of a historical site:

‘*Especially since P11 was, you know, completely fit and healthy one day, and, well, the same day, just suddenly, wallop. It was completely … changed P11, you know. So, yeah, it was a bit of a shock to the system’. (FM11).*

These family members discussed how, because of their shock and distress, and not really knowing what the patient’s preferences were, they had initially felt that they could not withhold any treatments that might have given them a chance of survival:

*‘So after two days of deterioration, so Doctor [name removed] said, what is your position on treatment and antibiotics; and I didn’t really have … I didn’t feel that I was in a … couldn’t not doing treatment. So I was trying to think about what would P12 say. P12’s really committed to life; so I said, well, I think if you felt it was okay I think P12 would want, wants to get better, P12’s not ready to die’. (FM12).*

Having initially asked for all treatments to be given, these family members reported how, over the days which followed this decision and as they got over their initial shock, they had reassessed the situation the patient was in and gathered evidence to make further decisions about (withdrawing) treatments. This included having discussions with family and friends about what the patient might have wanted with respect to treatments and future quality of life:

*‘And … initially my view was that because I didn’t have enough medical knowledge, I thought that feeding P11 and giving the antibiotics and the other medication, we would start to see an improvement. And, you know, I had a hope … whether it was a forlorn hope or not that the treatment would have an effect. But P11’s condition got worse- I’d spoken to various relatives and various friends of P11 and explained the situation and all of them said, oh P11 wouldn’t want to carry on living like that.’ (FM11).*

They also described how such discussions had jogged their memory about situations where the patient had previously made informal comments about life-extending treatments or surviving with disability. They then reported how these remarks had led them to conclude that the patient would not have wanted to have been kept alive by tube feeding or if they needed full-time care. For example, FM13 described how P13 had been the main carer for [gender of partner removed] who had had a stroke, and had asked that no life-extending treatments be given to [name and gender of partner removed]:

‘*I don’t think P13 would be very happy to be constantly fed and kept alive with tubes. My parent [gender removed] died with a stroke and P13 said the same thing, [gender of partner removed] wouldn’t want this, wouldn’t want that, wouldn’t be happy if couldn’t do XY and Z. So P13 was probably the most calm out of the whole family when [partner of P13] died.’ (FM13).*

Many also described how, when they were visiting the patient in hospital, they had observed them making gestures, such as removing oxygen masks and feeding tubes, which they interpreted as them wanting to reject these treatments:

‘*I think a lot of it was informed by the fact that P11 kept taking the feeding tubes out … And … just other signs. I mean, as P11’s family member [gender removed], I know P11’s facial expressions. And I just got the impression looking at P11 that P11 really wasn’t happy in the situation that P11 was in. P11’d had enough and wanted it come to an end. P11 wouldn’t want to be in a care home lying there, you know, effectively unable to do anything. And I think P11 was telling us that by removing the feeding tube and … telling us again by removing the oxygen’. (FM11).*

While reflecting on the situation, and realising that the patient might not survive the stroke, many of these family members described how they had moved away from their initial hope that the patient would recover to a more pragmatic approach of looking for potentially realistic information from the doctor on the patient’s likely (poor) prognosis. They then described how they used this information to decide on the appropriateness of (withdrawing) various life-extending treatments:

*‘Well each time a decision came along, I sat down with either Dr [name removed] or Dr [name removed] in the main and the main decision was on feeding and whether they should persist with it. So … yeah, I was given information. I asked them questions. We came to a judgment...’ (FM11).*

Although these family members described how, having reflected on the situation, they had decided that withdrawing treatments had been appropriate, they also noted how this process of decision-making (and treatment withdrawal) had been very upsetting for them. Some expressed how formal psychological support from hospital staff might have been helpful to them during this distressing time:

*‘You know, this is hard, very tough … some, kind of, counselling service available, preferably with people with some medical knowledge’. (FM11).*

#### Family members who had asked for all treatments to continue at all costs

The minority of family members, where patients had been young and independent before the stroke, described how they had felt shocked, overwhelmed and emotionally unprepared for the situation they now found themselves in. For example, FM14, the family member of a previously independent [patient gender removed] in [patient gender removed] 60s’, described how FM14 and [FM14’s parent- gender removed] had felt helpless and extremely distressed seeing P14 in hospital in a physically dependent and agitated state:

*‘I saw P14, my [parent- gender removed] was in shock basically. It was quite upsetting to see P14 being sick and looked like P14 was not comfortable. It just felt yesterday nobody was helping P14 to try and get this bleed under control and trying to get P14 back. So it’s, kind of, upsetting [sounding upset].’ (FM14).*

These family members expressed how, while feeling extremely distressed, they had looked for ways to maintain hope that the patient would survive. For example, FM14 described how FM14 thought back to instances in the past where P14, based on P14’s determination to improve, had recovered well from minor illnesses. FM14 expressed how FM14 felt that, based on these previous situations, the current situation P14 was in would be one from which P14 would be able to pull through:

*‘I think P14 would cope with a lot. P14 can cope with a lot. P14 did have an operation on P14’s arm and had to get a plate put in and they did say to P14 that P14 would only get … likely 45/50 per cent usage. But P14 pushed on and pushed on and got 90 per cent usage in P14’s arm. They say P14 would only manage to get arm to here [lifting arm up from the table]. P14 can actually get arm to there [extending arm to 60 degrees]. And, you know, P14’s a determined [patient gender removed].’(FM14).*

In a related example, FM15 described how FM15 had looked for information from the doctor that gave FM15 hope that P15 would survive:

‘*To have heard from the doctor when [doctor gender removed] had said to us, you know, some people will survive, kind of, gave us a bit of hope; like, well, there is hope.’ (FM15).*

In their situation of extreme anguish, they expressed how they thought that all treatments should be given to the patient to promote the possibility (however small) of them surviving the major stroke:

*‘When it’s a family member like you don’t want them to withdraw treatment, you want them to give a 100 per cent and keep going no matter what. If a patient needs to be fed through a tube then they need to be fed through a tube and I don’t think that’s a decision that should be given to the family. It should just be...it should just happen’. (FM15).*

These family members also expressed how they felt isolated at this difficult time and reported that emotional support would have been helpful:

*‘[FM14’s parent- gender removed]‘s not coping, we were just left, left like that. There’s no one … Some sort of support would have been helpful, you know … but there was nothing..’ (FM14).*

## Discussion

### Summary of key results

To our knowledge, this is the first qualitative study exploring early treatment decision-making by family members on behalf of patients with major stroke. Family members’ approaches towards treatment decision-making lay on a spectrum, based on the patient’s pre-stroke functional status and prior experiences of illnesses, and any views they had expressed about treatment preferences in the event of a critical illness which might result in significant disability or death.

At one extreme of the treatment decision-making spectrum, there were family members who had decided not to initiate life-extending treatments at stroke onset due to the patients’ deteriorating health pre-stroke and stated treatment preferences pre-stroke. These family members looked for information from doctors to justify, rather than arrive at, their treatment decision. In the middle of this spectrum were family members of patients who were relatively independent, who decided to withdraw treatments over time once they got over the initial shock of the diagnosis and had time to gather relevant information from family, friends and doctors. At the other end of the spectrum were family members of previously independent patients whose treatment preferences were unknown. These family members asked for all treatments to continue at all costs and reported the need for hope of patient survival from doctors and psychological support.

Below, we place these findings into context of existing literature, and make recommendations for clinical practice.

### The need to explore the patient’s state of health before stroke

Our results agree with sociological literature reporting that the experiences of health and illness of individuals and relatedly, treatment decision-making, are socially and contextually informed [40]. Our results also corroborate results from studies involving family members of patients admitted to intensive care which have reported that, in addition to information regarding prognosis from doctors, the majority of family members estimated the patient’s prognosis depending on their perceptions of the patient’s strength of character, unique story of illness and survival and previous experiences and choices of treatments [41, 42].

Family members who decided not to initiate life-extending treatments appeared to have already experienced some anticipatory grief [43] and seemed to be prepared for the possibility that the patient might not survive. During the decision-making process, they had drawn on their previous experiences with those of the patient (for example, of the patient’s multiple hospital admissions, and declining health and quality of life), and knowledge of the patient’s treatment preferences. In contrast, where family members were unaware of the patient’s preferences, they were generally in shock and unprepared for a situation of critical illness [19] and therefore, found treatment decisions more difficult to make [41]. Our study therefore further reiterates the need for doctors to explore the patient’s preferences by gathering information from family members, perhaps through a narrative approach, i.e. by developing the patient’s story [44].

### Providing tailored information

As we have reported, the type of information that a family member might need varied depending on the patient’s health state and stated preferences pre-stroke. Our findings therefore provide insight to doctors to help them better prepare for discussions about prognosis with family members. For example, before meeting with families of older and dependent patients, doctors can prepare themselves by ensuring that they can provide realistic information about the patient’s (likely poor) prognosis and perhaps discuss treatments to optimise comfort. A practical four step approach may be appropriate, where the doctor initiates the discussion, clarifies understanding of prognosis with family members, identifies end-of-life goals and collaboratively develops an appropriate treatment plan focusing on comfort and symptom control [45]. For families of (relatively) independent patients, several meetings may be needed to share sufficient and relevant information, discuss preferences, weigh up pros and cons of available treatments and then arrive at decisions [31, 46, 47]..

In contrast, before meeting with families of young, previously fit and independent patients, doctors can prepare in advance on how best to deliver information, and address the likely emotional response (e.g. profound shock) as a result of the diagnosis. For example, doctors may consider using the ‘SPIKES’ protocol used in oncology which provides a six step strategy for breaking bad news and dealing with emotional responses [48]. Specifically, approaches such as active listening, observation of non-verbal communication, choosing words that may not be perceived negatively, breaking down information into small pieces and offering another meeting at an agreed time may help families understand the situation of major stroke with poor prognosis better and help them cope with their emotions [48, 49]. Doctors should also consider how to balance the communication of hope with that of realism [50]. This can be complex [51]. Some family members may maintain a strong sense of hope that the patient may survive and recover despite accepting poor prognosis [52, 53]. Others may find hope by being overly optimistic about the patient’s prognosis [29, 52, 54] and may not wish to obtain realistic information [55]. In contrast, some individuals may find hope when doctors discuss preparations for possible death and optimising comfort at end-of-life [56]. This further highlights the need for tailored communication, and doctors may consider adapting some communication strategies used in intensive care to the major stroke setting. For example, the use of the phrase ‘hope for the best and prepare for the worst’ can help manage expectations [57]. Using ‘I wish’ statements (e.g. ‘I wish things were different’) may also acknowledge the limits of available options while expressing empathy in a situation which may be futile, or where individuals may have unrealistic hopes [57, 58].

### Exploring the need for psychological support

Our results indicate that the shock of stroke diagnosis and being involved in decisions not to continue life-extending treatments can be upsetting for family members [59–61]. There is evidence that the distress individuals may feel can linger for months or even years [62]. Therefore, when meeting with family members, doctors may consider exploring if they had support from friends and other family [63] and if they may wish counselling and emotional support from clinically trained staff (e.g. psychologists) to help reduce their distress [64, 65].

### Recommendations for clinical practice

Based on our findings, we recommend doctors communicating with family members of patients with major stroke to consider the following:

1. To gather evidence regarding the patient’s story using a narrative approach; specifically, on the patient’s functional status pre-stroke and any previously stated preferences for life-extending treatments.
2. To tailor their communication of information depending on the individual’s needs: i.e. information to confirm poor prognosis, that to facilitate shared decision-making or information to maintain hope (while being realistic and not offering false hope)
3. To be aware that family members may have unmet psychological and emotional needs which would need identified. Some may require more specialist input, e.g. clinical psychology.

### Strengths and limitations

We engaged with a group of family members at a time which can be emotionally distressing for them and therefore, we were able to gain novel and important insight into their experiences, needs and approaches towards treatment decision-making. However, our sample size was relatively small and homogenous; all participants were of similar ethnicities and were recruited from one tertiary teaching hospital. Furthermore, since this study relied on participants ‘opting-in’, it is possible that individuals who participated were those who were more able to voice their experiences. Therefore, our sample of family members may not be representative of all family members of patients admitted with major stroke which may reduce the transferability and generalisability of our findings to other populations [66]..

### Recommendations for further research

Future research could consider investigating the experiences, views and needs of families from different socio-economic and ethnic backgrounds, who may have different experiences and approaches towards treatment decisions. Staff training on communication strategies (e.g. using the ‘SPIKES’ protocol, ‘Hope for the best, prepare for the worst’) in major stroke would be helpful, and future research could consider further adapting and evaluating these strategies in the context of major stroke.

## Conclusions

We identified a spectrum of treatment decision-making approaches by family members of patients with major stroke, defined by the patient’s state of health and stated preferences pre-stroke, which influenced information and support needs of family members. The knowledge that such a spectrum exists may allow doctors to better prepare for discussions with family members regarding the patient’s prognosis. Therefore, they may be able to provide information and support that is tailored towards family members’ needs.

## Data Availability

All data generated or analysed during this study are included in this published article and are not publicly available. This is to safeguard participant confidentiality. Transcripts are anonymised. Original recordings have been destroyed.

## Abbreviations

FM: family member
P: Patient
